# Hospitalisations at the end of life: using a sentinel surveillance network to study hospital use and associated patient, disease and healthcare factors

**DOI:** 10.1186/1472-6963-7-69

**Published:** 2007-05-08

**Authors:** Lieve Van den Block, Reginald Deschepper, Katrien Drieskens, Sabien Bauwens, Johan Bilsen, Nathalie Bossuyt, Luc Deliens

**Affiliations:** 1 End-of-Life Care Research Group, Vrije Universiteit Brussel, Belgium; 2 Centre for Oncology, Academic Hospital Vrije Universiteit Brussel, Belgium; 3 Centre for Environmental Philosophy and Bioethics, Ghent University, Belgium; 4 Scientific Institute of Public Health, Department of Epidemiology, Brussels, Belgium; 5 Department of Public and Occupational Health, and EMGO Institute, VU University Medical Centre, Amsterdam, The Netherlands

## Abstract

**Background:**

Hospital deaths following several hospital admissions or long hospital stays may be indicative of a low quality of dying. Although place of death has been extensively investigated at population level, hospital use in the last months of life and its determinants have been studied less often, especially in Europe and with a general end-of-life patient population. In this study we aim to describe hospital use in the last three months of life in Belgium and identify associated patient, disease and healthcare factors.

**Methods:**

We conducted a retrospective registration study (13 weeks in 2004) with the Belgian Sentinel Network of General Practitioners, an epidemiological surveillance system representative of all GPs in Belgium, covering 1.75% of the total Belgian patient population. All registered non-sudden or expected deaths of patients (aged one year or older) at the GPs' practices were included. Bivariate and regression analyses were performed.

**Results:**

The response rate was 87%. The GPs registered 319 deaths that met inclusion criteria. Sixty percent had been hospitalised at least once in the last three months of life, for a median of 19 days. The percentage of patients hospitalised increased exponentially in the last weeks before death; one fifth was admitted in the final week of life. Seventy-two percent of patients hospitalised at least once in the final three months died in hospital. A palliative treatment goal, death from cardiovascular diseases, the expression of a wish to die in an elderly home and palliative care delivery by the GP were associated with lower hospitalisation odds.

**Conclusion:**

Hospital care plays a large role in the end of patients' lives in Belgium, especially in the final weeks of life. The result is a high rate of hospital deaths, showing the institutionalised nature of dying. Patients' clinical conditions, the expression of preferences and also healthcare characteristics such as being treated as a palliative care patient, seem to be associated with hospital transfers. It is recommended that hospitalisation decisions are only made after careful consideration. Short admissions in the final days of life should be prevented in order to make dying at home more feasible.

## Background

Long or repeated hospital admissions at the end of life and death in a hospital setting could be an indicator of a low quality of dying [1-3]. Although some end-of-life hospitalisations are necessary and could benefit the patients, patients express an overall preference to die at home in the presence of their loved ones [4,5]. Additionally, aggressive life-prolonging and possibly futile interventions, often in opposition to the palliative care needs and preferences of patients at the end of their lives, are more likely to be carried out in hospitals, and patients in institutions have higher rates of unmet physical and psychosocial needs than patients dying at home [6-8]. Reduction of the time spent in the hospital and of the number of hospital admissions is therefore an important issue in palliative care [9,10].

In several countries, including Belgium, death has become institutionalised, with most people dying in hospital [11-13]. Although place of death and its determinants has been extensively investigated [11,12,14-16], hospital use and transitions between care settings in the last months of life and its determinants have been studied less often. This is especially the case in Europe and with a general end-of-life patient population. Studies that investigate hospital use at the end of life are often limited to specific diagnoses (e.g. cancer) [9,17], age groups (e.g. the elderly) [18-21], or settings (e.g. hospital, nursing homes or specialist palliative care services) [22,23] and they focus on specific determinants of hospital use (e.g. hospice involvement) [10,22].

Analyses of the timing and nature of hospital use at the end of life can provide important information on the opportunities and challenges faced in the planning and implementation of healthcare services for dying patients. This could enable more people to be cared for and to die where they want to.

The purpose of this study is to describe hospital use in the last three months of patients' life, regardless of the place or cause of death, and to identify patient, disease and healthcare factors associated with hospital use. In this study, a general end-of-life patient population was identified over a three-month registration period by a Sentinel Network of General Practitioners in Belgium [24]. This is the first study reporting on hospital use at the end of life in Belgium. Our specific research questions were:

How many patients are hospitalised during the last three months of life, and how often does this occur?

How many days do patients spend in the hospital and in what period during the last three months of life?

What patient, disease and healthcare factors are associated with hospital use during the last three months of life?

## Methods

### Study design

The data for this study was collected by the Belgian Sentinel Network of General Practitioners (GPs), a weekly registering network operational since 1979 under the authority of the Scientific Institute of Public Health in Belgium. The network has proved to be a reliable surveillance system for health-related epidemiological data [24]. Its participants are representative of the profile of family physicians in Belgium, i.e. in terms of age, sex and homogeneous geographical distribution [25]. The network covers 1.75% of the total Belgian patient population.

We conducted a retrospective registration study with this network, including all deaths of patients aged one or older, occurring between April and June 2004 (13 consecutive weeks) as well as the last death before this period. To shorten the time between death and registration – hence preventing recall bias as much as possible – the physicians were instructed to register all deaths, immediately after being informed about the patient's death, on a continuous basis during the months of inclusion. To optimise accuracy of the data registered, they were also instructed to use patient records and information coming from hospital physicians as much as possible.

In order to identify a general end-of-life population [26,27], additional inclusion criteria for this study were patients who were part of the GP's (group) practice and whose death was labelled non-sudden or expected.

Several control measures (such as data-entry with consistency, range and skip checks, possibility of contacting GPs by phone, double data-entry) were used to ensure data quality and to prevent missing data.

The anonymity of the patient and the physician was preserved. The protocol of the study was approved by the Ethical Review Board of the University Hospital of the Vrije Universiteit Brussel.

### Questionnaire

The network GPs filled in a structured, standardized questionnaire for each death case. The first part surveyed the patient's date of birth and death, sex, overall socio-economic status (estimated by GP), the postal code of their habitual residence and the cause of death (encoded into ICD-10 codes). For all non-sudden deaths of patients that were part of the GP's (group) practice, a second part was filled in measuring characteristics of the last three months of life:

- place of death and places of residence including lengths of each hospital stay

- patient's wish concerning the place of death if expressed to the GP: "Were you informed (verbally or in writing) of the patient's preference with regard to their place of death?" And if so, "where did this patient wish to die?"

- continuity and GP support variables:

◦ GP's palliative care delivery: "Did you provide palliative care to this patient?" Yes (until death or not until death) or No.

◦ Number of GP contacts with the patient or relatives, concerning the patient (home visits or consultations, excluding telephone contact)

- social support variables: living situation, informal care delivery ("how often were partner, children, or other people actively involved in the care provided to this patient?")

- main focus of treatment (palliative versus curative/life-prolonging)

- involvement of specialist palliative home care and/or elderly home care teams.

The number of GP contacts, informal care delivery and the focus of treatment were measured in three separate time frames (the final week, second to fourth week and second to third month before death).

### Data analysis

We calculated the proportion of patients hospitalised for at least one day during the last three months of life and the number of times they were admitted to the hospital in this period. The total number of days spent in hospital was calculated by summing the lengths of stay of all admissions. A stay in a specialist palliative care unit was not considered a hospital stay because of the large differences in cultures (care versus cure) between the two settings. In a limited number of cases, hospitalisation decisions made at home might be deflected to a stay in a palliative care unit, but GPs would probably be informed of this by the specialist caregivers in these units.

We calculated mean (standard deviation) and median (interquartile range) length of hospital stay in days for all patients admitted to a hospital for at least one day. To explore time trends in hospital use, we described when patients were admitted to the hospital and how many died there. We also calculated the hospitalisation rate defined as the proportion of patients who were in a hospital on day X before death.

We structured the possible determinants of hospital use at the end of life (patient, disease and healthcare characteristics) based on previously published models on the determinants of healthcare use and place of death [5,28,29]. We used Pearson Chi^2 ^tests to explore bivariate associations between these characteristics and the fact of being hospitalised for at least one day in the final three months of life or not being hospitalised, and logistic regression to calculate adjusted odd-ratios.

To explore factors associated with the length of hospital stays, the nonparametric analysis of variance (Kruskal Wallis test) was used in the bivariate analyses and a linear regression model in the multivariate analyses (the dependent variable in the model, number of hospital days, was logarithmically transformed to achieve a near-normal distribution). The independent variables measured in three time frames were concatenated to an overall score during the three final months of life. For example, treatment goal – measured in the last week, second to fourth week and second to third month before death – was concatenated to "curative/life-prolonging in last three months" or "palliative in last three months", or "from curative/life-prolonging to palliative in the last month or week of life". We used the postal codes of patients' residence to calculate the local degree of urbanization and ratio of hospital beds per 1000 inhabitants, using data from various ecological healthcare statistics available in Belgium. Analyses were performed using SPSS13.0.

## Results

Of the panel of 202 GP practices participating in the Sentinel Network in 2004, 176 (= 87%) reported one or more deaths. In total 502 deaths were reported, of which 332 were part of the GPs' (group) practices and died non-suddenly or expectedly. Thirteen cases were subtracted due to incomplete care trajectories. Hence the results of this study are based on 319 non-sudden deaths.

Age, sex, cause and place of death of these non-sudden deaths in the Dutch-speaking part of Belgium (n = 191) were compared with the non-sudden deaths identified in another study (2001) on end-of-life decisions representative for all deaths in this part of the country (n = 2128) [30]. The percentages of these characteristics in both groups showed no significant differences (using Multinomial 95% Confidence Intervals, exact method; data not shown). No comparison data was available for the French-speaking part of the country (i.e. 40% of the Belgian population).

### Number of hospitalisations, length and timing of hospital stay

**Figure 1:**
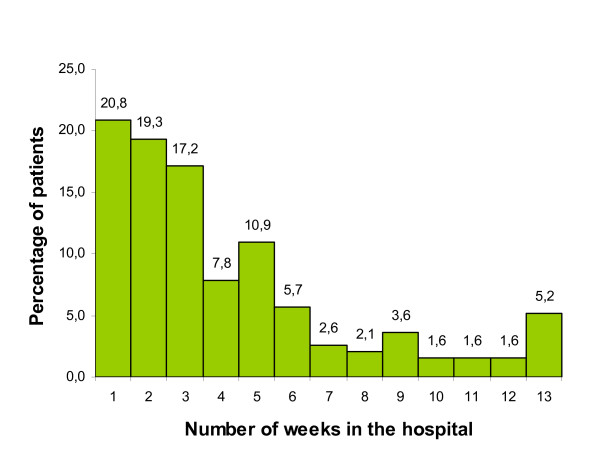
Total length of stay (weeks) in the hospital in the last three months of life (n = 192).

Table 1 shows that 60% of the patients stayed in a hospital for at least one day in the last three months of life. One fifth of this group were admitted to the hospital twice or three times in this period. The median length of hospital stay was 19 days. Figure 1 shows the variation in length of hospital stay in detail. One fifth of the patients had short hospital stays of one week or less. A majority of patients (57%) was hospitalised for three weeks or less.

**Table 1:** Hospital use in the last three months of life (N = 319) *

		**N (%)**
Not hospitalised		127 (39.8)
Hospitalised at least one day		192 (60.2)
	Hospitalised once	155 (80.7)
	Hospitalised twice	36 (18.8)
	Hospitalised three times	1 (0.5)
*If hospitalised in the last three months of life (N = 192):*		
Mean [sd] length of hospital stay in days		26.2 [23.9]
Median [interquartile range] length of hospital stay in days		19 [9 – 35]
Last hospital admission [number (%) of hospital deaths]	in last week before death:	34 (17.7) [33 (97.1)]
	in second to fourth week before death:	67 (34.9) [52 (77.6)]
	in second to third month before death:	91 (47.4) [53 (58.2)]
Death in hospital		[138 (71.9)]

* Within this population, we observed no differences between the reported deaths occurring between April and June of 2004 and the last death before this period regarding number of hospitalisations, length nor timing of hospital stay at the end of life.

Eighteen percent of hospital admissions took place in the week before death, 35% in the preceding three weeks and 47% in the preceding months (Table 1). All but one of the patients who were admitted in the final week of life died there. Overall, 72% of the patients hospitalised for at least one day in their last three months died in hospital.

**Figure 2:**
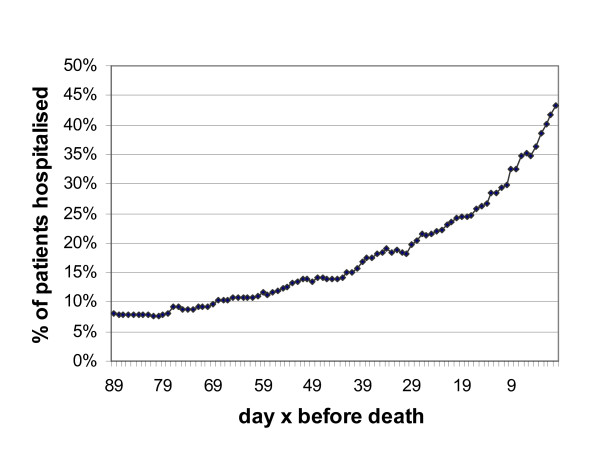
Hospitalisation rate in the last three months of life (n = 319).

Figure 2 shows the proportion of patients who were in a hospital on day X within the last three months before death. The percentage of patients hospitalised increased gradually in months three and two before death and exponentially as they got closer to death. Three months before death, 8% were hospitalised, increasing to 18% one month before death and 43% on the last day of life (= place of death). Patients who only stayed in hospital for short lengths of time (e.g. one to seven days) were mainly hospitalised in the final week (data not shown).

### Patient, disease and healthcare factors associated with hospital use

Table 2 lists the proportion of patients hospitalised or not in the last three months of life for subgroups of patients defined by patient, disease and healthcare characteristics. Hospital admission occurred most often among patients older than 65 and younger than 85 years old. Males were more often admitted than females, and patients whose wish for place of death was not known by the GP and patients who expressed a wish to die in a hospital or palliative care unit, were more often hospitalised.

**Table 2:** Patient, disease and healthcare factors associated with hospital use in the last three months of life (n = 319)

			**Not hospitalised** **(n = 127)**	**Hospitalised at least one day** **(n = 192)**	p-value*
**PATIENT AND DISEASE FACTORS**		**Total N †**	**%**	**%**	

Age at death	1–64 y	42	42.9	57.1	**.008**
	65–74 y	74	29.7	70.3	
	75–84 y	109	34.9	65.1	
	+85 y	91	53.8	46.2	
Sex	male	164	31.7	68.3	**.003**
	female	155	48.4	51.6	
Socio-economic status	low	77	41.6	58.4	.90
	medium	205	39.0	61.0	
	high	31	41.9	58.1	
Degree of urbanization §	core of large city	108	42.6	57.4	.33
	high	71	31.0	69.0	
	average	76	40.8	59.2	
	low or rural	62	45.2	54.8	
Place of death wish expressed to GP	home or living with family	96	54.2	45.8	**<.001**
	home for the elderly	37	73.0	27.0	
	elsewhere (hospital, palliative care unit)	17	23.5	76.5	
	not expressed	157	23.6	76.4	
Cause of death (ICD-10)	malignancies	124	35.5	64.5	.40
	cardiovascular diseases	83	44.6	55.4	
	diseases of the nervous system	13	53.8	46.2	
	other	97	38.1	61.9	

**HEALTHCARE FACTORS**					

Hospital beds in healthcare area (rate/1000 inhab) ‡	<5.658	185	43.2	56.8	.16
	>=5.658	133	35.3	64.7	
CONTINUITY AND GP SUPPORT					
Palliative care delivered by GP in last three months	yes	146	61.6	38.4	**<.001**
	no	171	20.5	79.5	
Total number of GP contacts in last three months (consultations, home visits)	low [0–6]	113	26.5	73.5	**.001**
	medium ]6 – 11]	105	44.8	55.2	
	high ]11 – 88]	101	49.5	50.5	
SOCIAL SUPPORT					
Living situation §	at home, with partner and other adults:	51	54.9	45.1	**.003**
	at home, with partner but no other adults:	115	30.4	69.6	
	at home, single or with children aged <18 y:	51	31.4	68.6	
	living elsewhere without family: ∥	95	49.5	50.5	
Informal care (partner, child, acquaintance,...) in last three months	none or very little	111	36.0	64.0	.46
	yes, sometimes	133	43.6	56.4	
	yes, often	49	42.9	57.1	
INVOLVEMENT OF SPECIALIST PALLIATIVE CARE					
Specialist palliative home care team	yes	37	54.1	45.9	.11
	no	260	38.8	61.2	
Specialist palliative elderly home care team	yes	21	76.2	23.8	**.001**
	no	276	38.0	62.0	
FOCUS OF TREATMENT					
Treatment goal in last three months	curative/life-prolonging:	79	12.7	87.3	**<.001**
	palliative:	76	64.5	35.5	
	from curative/life-prolonging to palliative:	130	42.3	57.7	
	from palliative to curative/life-prolonging: ¶	2	-	100.0	

* p-value for all variables (Pearson χ^2^-test); significant values are bold

† missing values (not responded or unknown) not included in bivariate analyses: for age n = 3; for socio-economic status n = 6; for degree of urbanization n = 2; for patient's wish for place of death n = 12; for cause of death n = 2; for hospital beds rate n = 1; for palliative care delivered by GP n = 2; for living situation n = 7; for informal care n = 26; for specialist palliative caren = 22; for treatment goal n = 32

‡ measured on level of care districts or provinces, dichotomised at their median values; §on the basis of patient residence

∥ 87.4% of this group are elderly home residents

¶ not included in analyses

Abbreviations: GP = General Practitioners

With respect to healthcare characteristics, hospitalisation rates were higher where GP support was lower, i.e. if the GP reported not having delivered palliative care or if s/he had had a low number of contacts with the patient or relatives. Social support was also related to hospitalisation, with patients living at home alone, with children under 18 years old or alone with a partner, more often hospitalised, than patients living at home with a partner and other adults, or in an institution. Involvement of specialist palliative care in the elderly homes and a treatment with a palliative (rather than curative or life-prolonging) focus, was associated with a lower percentage of hospitalised patients.

While many characteristics were related to hospital admission, few were associated with the length of hospital stay (data not shown). In general, the differences in averaged or median length of stay for the subgroups of patients were small. Lower averages of length of hospital stay were found for patients receiving palliative care from the GP (p = .022) and for patients whose treatment focus was palliative (p = .013).

Multivariate logistic regression analyses (Table 3) shows that the odds of being hospitalized were four times lower if a wish to die in an elderly home was known to the GP. Patients who had expressed a wish to die at home had the same chance of being hospitalised as patients whose wishes were not known. If the GP provided palliative care, his/her patients had a four times lower likelihood of being hospitalised. Cause of death was not retained in the bivariate analyses, but, if adjusted for the other characteristics, patients with malignancies were more often hospitalised than those with cardiovascular diseases (three to four times more often) and equally often as those with diseases of the nervous system. Finally, a curative goal of treatment during the entire last three months of life increased the odds of being hospitalised five times, and patients whose treatment goal was changed from cure or life-prolonging to palliative care in the final month or week of life had a chance of hospitalisation that was twice as high as for patients whose treatment had been focussed on palliative care during the entire last three months of life. If patients whose treatment goal changed were hospitalised, this usually occurred before their final month of life (data not shown).

**Table 3:** Logistic regression analyses of factors associated with hospital use in the last three months of life (n = 319) *

		**Beta**	**Standard Error**	**Odds-ratios [95%CI] †** **Hospitalised or not**
Place of death wish expressed to GP	not expressed:			1.00 [1.00-1.00]
	home or living with family:	-0.603	0.348	0.547 [0.277–1.083]
	home for the elderly:	-1.404	0.493	**0.246 [0.093–0.645]**
	elsewhere (hospital, palliative care unit):	0.263	0.678	1.301 [0.345–4.908]
Palliative care delivered by GP	no			1.00 [1.00-1.00]
	yes	-1.405	0.366	**0.245 [0.120–0.503]**
Cause of death (ICD-10)	malignancies			1.00 [1.00-1.00]
	cardiovascular diseases	-1.305	0.390	**0.271 [0.126–0.582]**
	diseases of the nervous system	-0.284	0.902	0.753 [0.129–4.411]
	other	-1.053	0.374	**0.349 [0.168–0.726]**
Treatment goal in last three months before death ‡	palliative:			1.00 [1.00-1.00]
	curative/life-prolonging:	1.686	0.517	**5.398 [1.958–14.882]**
	from curative/life-prolonging to palliative:	0.830	0.353	**2.293 [1.147–4.584]**

* Multivariate logistic regression analyses for hospitalised (at least one day) (1) versus not hospitalised (0) patients; Missing values were included as a separate category if N>20; Variables entered in the regression were all variables significant in the bivariate analyses and cause of death, hospital beds in the healthcare area, degree of urbanization, and informal care provided [entered on the basis of results of other research e.g. (3)]. Interaction effects of the remaining variables were entered in the regression but not significant. No problems of multi-collinearity were observed.

† Odds-ratios with 95% Confidence Intervals are displayed; significant relationships are bold; Model summary results: Nagelkerke R Square = 0.368; Percentage correctly predicted = 76%;

‡ the subcategory "from palliative to curative/life-prolonging (n = 2) was not included in analyses

Abbreviations: GP = General Practitioners, ICD-10 = International Classification of Diseases version 10

No multivariate linear regression model could be constructed for length of hospital stay because no entered variables were retained.

## Discussion

Hospital care plays a large role in patient care at the end of life. In Belgium, two out of three dying patients are hospitalised at least once during the final three months. The average patient spends approximately three weeks in hospital, but a large inter-individual variation in length of stay could be observed. The proportion of hospitalised patients increased exponentially in the last month before death – one fifth were admitted in the last week – with a high rate of hospital deaths as a result. A palliative treatment goal, death from cardiovascular diseases, expression of a wish to die in an elderly home and palliative care delivery by the GP were associated with a lower likelihood of being hospitalised in the last three months of life.

In this study, we were able to measure hospitalisation and associated patient, disease and healthcare characteristics in the final three months of life in a general sample of non-sudden deaths, irrespective of diagnoses or care setting. This was possible via the Belgian Sentinel Network of GPs because the network is representative of the GP profile in Belgium [25] and almost all of the population (95%), including elderly home residents, have a regular GP, who is easily accessible and consulted on a regular basis [31]. The network has proved to be a reliable surveillance system for health-related epidemiological data covering 1.75% of the total Belgian patient population [24,32]. The non-sudden deaths identified in this study – in the Dutch-speaking part of the country, i.e. 60% of the population – were judged representative for all non-sudden deaths in this part of the country [30]. We could gather high quality data because co-operation of the GPs in the network is optimal, because they are familiar with scientific registration and because quality control measures were used [24,25].

There were also some limitations, however. Firstly, because the observational unit of this study was the GP, we did not evaluate subjective states such as patients' quality of life or symptom burden which might have further explained hospital transfers. Secondly, the retrospective design of the study could have induced recall bias among the GPs and therefore could have influenced the results. Retrospective reconstructions of the care provided might also deviate from the actual care given. Finally, relationships between cause and effect could not be established in our study: we could only explore associations between characteristics and hospitalisation.

The results of this study show the institutionalised nature of the final phase of life in Belgium. The high frequency of hospital transfers and high hospital use emphasises the fact that acute care hospitals play a large part in meeting the needs of dying patients, with an exponential rise in the last month of life leading to a high proportion of hospital deaths. The organization of healthcare in Belgium seems clearly oriented towards specialist care provision with a low threshold for specialist hospital care. Research in specific patient populations in Canada [17], the US [22] and Europe [9,23] is consistent with this study, i.e. increasing hospitalisation rates during the last month of life, but detailed comparisons are difficult mainly because other inclusion criteria were used such as cancer patients, patients in hospitals or specialist palliative care.

An interesting finding is that if a patient is hospitalised at some time during the final 3 months of life, the chance of dying in a hospital is very high: 72% of patients hospitalised at least once died in a hospital. Since few patients have multiple transfers to a hospital and many hospitalisations occur in the final days or weeks before death, this finding suggests that for many patients the decision to hospitalise is tantamount to the decision to die in the hospital.

Several patient, disease and healthcare characteristics are closely related to hospital admissions. It is striking that not only patients' clinical condition (e.g. cancer) is indicative for whether or not they are hospitalised, but that a treatment focused on palliative care instead of life-prolongation or cure considerately lowers the chance of being hospitalised. This is consistent with one of the goals of palliative care, which is to reduce hospitalisation rates at the end of life, and also with the cure-oriented culture attributed to hospital settings [9]. Being a "palliative care patient" seems to be an important consideration when making transfer decisions. Unfortunately, palliative care is often started too late [33] and the survival time of dying patients is often overestimated [34], possibly leading to a high number of hospitalisations at the end of life.

Patients who have expressed a wish for home death were hospitalised as often as patients whose wish was not known to the GP, but elderly home residents who expressed a wish to die there were hospitalised less often. A possible explanation is the presence of professional caregivers in elderly homes which makes it possible to carry out their wishes, while patients at home often depend on informal caregivers who possibly cannot provide the complex care needed for extended periods of time at the end of life [28,34]. The heavy burden on GPs who treat dying patients at home might also explain why patients' wishes for home death are carried out less often than those of elderly home patients. In both settings, wishes might also have been expressed after a first hospitalisation and consequently prevented a second admission, which would further strengthen the relationship between expression of wishes and hospitalisation. However, this cannot be determined from our data and should be studied in future research.

The GP might also play an important role in the hospitalisation decision. If the GP perceived that he or she delivered palliative care to the patient, the odds of being hospitalised were smaller. In accordance with studies on GPs' palliative care delivery, this could mean that a lack of GP involvement in palliative care, often related to a lack of confidence in their own palliative care skills [35], leads to high hospitalisation rates. However, hospitalisation itself might be the reason why GPs' involvement in palliative care delivery was lower. Furthermore, the GP might have made a retrospective reconstruction of his/her involvement based upon what actually happened at the end of the patient's life. This relationship should be investigated in more detail in future research before definite conclusions can be drawn.

The involvement of multidisciplinary palliative home or elderly home teams supporting the GP seems to have no association with hospital admission, which is inconsistent with some results in other studies [10]. A possible explanation is the late onset of these specialized healthcare services [33], often during the last week of life. An effect on hospitalisation rates can only be measured after their initialisation, while we focused on a three-month period in this study. Furthermore, patients might have been hospitalised before their involvement. Future research should register the timing and intensity of their involvement in patient care.

Finally, a key question is whether end-of-life hospitalisations are necessary and whether they benefit the patient. Some hospitalisations are indeed necessary, especially if a specific hospital treatment is needed e.g. in the case of a 60-year-old patient who was admitted for curative chemotherapy 3 months before death, when cure was still believed possible. Also, the increase in complex medical problems at the end of life makes them difficult to manage at home or in an elderly home. On the other hand, the necessity of many hospitalisations at the very end, especially those short stays in the final weeks of life, as well as admissions of this fragile population for long periods of time, could be questioned. Most patients do not want to die in a hospital [4]. Transferring them at the end of their life might be extremely stressful, since their caregivers and environment would change abruptly [36]. Hospital care is often aggressive, which might oppose to the palliative care needs of many dying patients, especially at the very end of life [6-8]. Also, several problems with continuity of care might arise from these transfers [36] such as information concerning patient wishes to receive or not receive specific treatments (e.g. do-not-resuscitate orders) might not be transferred. Of course, acute situations cannot be fully prevented. However, even then, adequate advance care planning, taking into account patient preferences, remains important.

## Conclusion

In Belgium, dying is highly institutionalised. Hospital transfers rise in the last weeks before death, leading to a high rate of hospital deaths. Prevention of short admissions close to death will facilitate dying at the place of wish. Patients' clinical conditions and preferences as well as several healthcare characteristics such as the patients' treatment focus or the involvement of their primary care physicians, are associated with hospital transfers. The extent to which these factors might improve quality of end-of-life care by preventing unnecessary hospital admissions and facilitating dying at the place of wish, needs to be studied in future research.

## Competing interests

The author(s) declare that they have no competing interests.

## Authors' contributions

LVDB, RD, SB and LD were involved in the conception and design of the study. LVDB, KD and NB gathered the data. Statistical analyses were carried out by LVDB and critically revised by RD, JB, NB and LD. The manuscript was drafted by LVDB. LD was the project supervisor. NB is part of the coordinating centre of the Sentinel GP Network. All authors have made major contributions to data interpretation and critical revision of the manuscript. They have all read and approved the final manuscript.

## Pre-publication history

The pre-publication history for this paper can be accessed here:


